# Mechanism, Diagnostic and Therapeutic Novelties in Dermatology: Where Are We Going?

**DOI:** 10.3390/life14101236

**Published:** 2024-09-27

**Authors:** Ricardo Ruiz-Villaverde, Marta Cebolla-Verdugo, Alvaro Prados-Carmona, José Juan Pereyra Rodríguez, José Carlos Armario-Hita

**Affiliations:** 1 Hospital Universitario San Cecilio, 18016 Granada, Spian; martacevers@gmail.com (M.C.-V.); apradoscar@gmail.com (A.P.-C.); 2 Instituto Biosanitario Ibs Granada, 18014 Granada, Spain; 3 Hospital Universitario Virgen del Rocío, 41013 Sevilla, Spain; pe3reyra@gmail.com; 4 Hospital Universitario Puerto Real, 11510 Cádiz, Spain; jcarmarioh@gmail.com

Dermatology is one of the oldest specialties and yet one of the most innovative and constantly evolving ones, too. It is a fairly self-sufficient specialty, as the accessibility of the skin, mucous membranes, appendages, etc., allows a diagnosis without resorting to complementary means on many occasions. However, there is currently a real revolution in the understanding of the pathophysiological mechanisms of different inflammatory and tumoral skin diseases, in the complementary techniques that we use to develop diagnosis (ultrasonography, dermoscopy, confocal microscopy, etc.) and in the availability of alternative approaches to treatment that use expensive biological drugs and synthetic molecules. Therefore, it seems appropriate to review the topics developed in this first edition and what conclusions can be drawn to apply to our daily clinical practice.

Sulzbach Denardin M et al. [1] analyzed 785 polymorphisms from 144 genes related to histone (de)acetylation among endemic pemphigus foliaceus (EPF) patients and controls. This study found that certain genetic variants in HDAC4, GSE1 and PHF21A are associated with EPF susceptibility, while these associations were absent in a German cohort with sporadic PF (SPF), suggesting different underlying mechanisms for EPF and SPF. Specifically, individuals carrying the HDAC4_rs4852054A allele had a higher risk of developing EPF, while those with GSE1_rs13339618A and PHF21A_rs4756055*A showed reduced susceptibility. In addition to genetic analyses, the study examined the RNA expression of these genes in CD4+ T lymphocytes. Key findings include the upregulation of KAT2B, PHF20 and ZEB2, and the downregulation of KAT14 and JADE1 in patients with active EPF. These genes are involved in epigenetic regulation and may influence immune cell differentiation and apoptosis, contributing to the disease’s progression. The results suggest that histone acetylation and deacetylation play critical roles in EPF’s autoimmune response, influencing the immune system’s tolerance mechanisms. This study provides insights into the potential epigenetic factors involved in EPF and highlights the importance of further research on chromatin state alterations in autoimmune diseases like PF.

Trichology is a growing field in the dermatological world; within this field, scarring alopecia and especially frontal fibrosing alopecia continues to be a real challenge. Frontal fibrosing alopecia (FFA) is a type of scarring alopecia primarily affecting postmenopausal women, characterized by progressive hairline recession. Despite its rising incidence, the exact pathogenesis remains unclear, with hormonal, genetic, autoimmune and environmental factors being implicated. The study of Carmina-Rodriguez et al. [2] has analyzed 306 patients over an 11-year period, providing valuable clinical insights into FFA, its progression, and associated conditions. FFA is associated with autoimmune disorders, with hypothyroidism being common among patients. The study found a statistically significant correlation between disease severity and progression time, with longer disease duration predicting more severe hair loss. Interestingly, the presence of inflammatory trichoscopic signs such as erythema and hyperkeratosis was not associated with disease severity, suggesting these indicators may not reliably predict progression. Eyebrow alopecia, observed in 90.5% of patients, was linked to milder forms of the disease, offering a potential early diagnostic clue. In clinical practice, the early recognition of FFA is critical to managing progression and minimizing disfiguring hair loss. The findings suggest that while FFA can be difficult to treat, systemic therapies such as 5-alpha reductase inhibitors (e.g., dutasteride) appear to be the most effective at stabilizing disease progression. However, no current treatments can reverse scarring or induce regrowth in affected areas, emphasizing the importance of early diagnosis.

Skin oncology and its etiopathogenic factors have also been included in this Special Issue. Drexler K [3] explores the relationship between actinic elastosis, a marker of chronic ultraviolet (UV) exposure, and the development of keratinocyte cancers (KC), specifically cutaneous squamous cell carcinoma (cSCC) and basal cell carcinoma (BCC). The study aimed to determine whether prolonged sun exposure correlates more strongly with one cancer type than the other, offering insights that are crucial for clinical practice. A key finding is that the severity of actinic elastosis is significantly higher in cSCC than in BCC, regardless of factors such as age, sex and tumor location. This suggests that cSCC is more closely linked to chronic UV radiation, while BCC is associated with intermittent UV exposure. A semiquantitative scoring system to assess actinic elastosis is introduced, demonstrating that routine histology can be used to estimate a patient’s lifetime UV exposure, which is particularly relevant for diagnosing and managing skin cancers. In clinical practice, understanding these distinctions is vital. Patients with a history of prolonged sun exposure, especially those presenting with signs of actinic elastosis, should be monitored closely for cSCC. This also highlights the importance of early intervention and preventive measures, such as sun protection and education about UV risks, especially for outdoor workers who are more likely to develop cSCC.

Regarding therapeutics, the study conducted by Velasco-Amador et al. [4] investigates the effectiveness and patient satisfaction associated with low-dose UVA1 phototherapy for various dermatological conditions in a single-center setting in Spain. The research involved 78 patients, with 46 completing the treatment protocol. The findings revealed an impressive overall clinical response rate of 91.3%, with complete responses observed in 36.96% of cases, particularly among patients with morphea and chronic hand eczema. Additionally, patient satisfaction, assessed using the TSQM-9 scale, was notably high, especially in conditions such as mastocytosis and systemic sclerosis. UVA1 phototherapy has emerged as a promising therapeutic option for a range of inflammatory and sclerotic skin diseases, demonstrating efficacy in treating conditions like prurigo nodularis and dyshidrotic eczema. The study highlights that both low and high doses of UVA1 are effective with no significant differences in the outcomes, suggesting that low-dose treatments can be a viable alternative. The therapy is generally well-tolerated with only minimal side effects being reported, making it an attractive option for patients. The implications of this study are significant for clinical practice, as they underscore the potential of low-dose UVA1 phototherapy in managing challenging dermatological conditions. The high response rates and patient satisfaction indicate that this treatment could enhance the quality of life for patients suffering from chronic skin diseases. However, the study acknowledges its limitations, emphasizing the need for further research to optimize treatment protocols and better understand patient variability in response.

A comprehensive examination of the relationship between advanced glycation end products (AGEs) and skin quality across various age groups, highlighting the significant implications for clinical practice in dermatology and aging research, has also been presented in this Special Issue. Conducted by Martinovic et al. [5] with a cohort of 237 participants, the study reveals critical correlations between AGEs and several skin parameters, including melanin, erythema and transepidermal water loss (TEWL). Notably, the findings indicate that higher levels of AGEs are associated with increased melanin and erythema, as well as elevated TEWL, while demonstrating negative correlations with skin hydration and friction. The research underscores the role of AGEs in skin aging, suggesting that they may disrupt skin barrier function and hydration, which are essential for maintaining skin health. The study’s multiple linear regression analysis identifies age, melanin, erythema and TEWL as positive predictors of AGEs, whereas hydration and friction serve as negative predictors. These insights are particularly relevant for clinicians, as they emphasize the need for targeted interventions that address the accumulation of AGEs to improve skin quality and mitigate the effects of aging. In this case, the authors highlight the importance of understanding the molecular mechanisms of glycation and its impact on skin properties, which can inform the development of effective anti-aging treatments.

Dodero-Anillo et al. [6] addresses the critical issue of optimizing therapeutic intervals for biologic drugs in the management of psoriasis, a chronic inflammatory skin condition that significantly impacts patients’ quality of life. The study highlights that a substantial proportion of patients do not achieve a complete response to biologic therapies, and how the efficacy of these treatments can diminish over time. Through a retrospective observational study, the authors analyzed plasma levels of three commonly used biologics—Infliximab (IFX), Adalimumab (ADL) and Etanercept (ETN)—alongside anti-drug antibodies to establish new therapeutic ranges.

The findings have revealed a significant correlation between subtherapeutic drug levels and treatment failure, underscoring the importance of therapeutic drug monitoring in clinical practice. Specifically, the study proposes new therapeutic ranges: 2–10 μg/mL for IFX, 3–11 μg/mL for ADL and 1–7 μg/mL for ETN. These revised ranges enhance the sensitivity of detecting treatment responses, suggesting that current therapeutic thresholds may be excessively high. This research is pivotal for clinical practice as it emphasizes the necessity of individualized treatment approaches based on drug monitoring. By adjusting therapeutic ranges, clinicians will be able to better predict treatment outcomes and optimize patient management strategies.

The elderly population is often an undervalued target population in clinical trials and so it is an interesting demographic to extract scientific evidence from; a study contained in our Special Issue uses this subject area in a large sample size case series. This manuscript [7] emphasizes the significance of contact hypersensitivity (CH) in older adults, a condition that becomes more prevalent as the elderly population grows. Aging leads to both intrinsic and extrinsic changes in the skin and immune system, such as decreased skin barrier function and impaired wound healing. These changes make the elderly more susceptible to allergens, despite a declining immune response. The research, which involved 600 elderly patients, found that over half (54.8%) exhibited at least one positive patch test, indicating contact allergy. The most common allergens identified were benzoic acid, methylisothiazolinone (MI), wood tar, nickel and balsam of Peru. The study highlighted that while nickel allergy is more commonly acquired earlier in life, preservatives like MI and benzoic acid were prevalent allergens in the elderly. Notably, females had a higher prevalence of CH than males (78.7% vs. 21.3%). The clinical relevance of this study lies in its recommendation to conduct patch testing for conditions like contact dermatitis, stasis dermatitis, rosacea and atopic dermatitis in patients over 60. These findings are crucial for daily clinical practice, as early identification of contact allergens can improve the treatment outcomes and quality of life in elderly patients. By pinpointing prevalent allergens, clinicians can better manage contact dermatitis and associated comorbidities in this age group, allowing for tailored, preventive measures in dermatological care.

Nevertheless, treatment using a CO_2_ laser for lichen sclerosus et atrophicus has led to a revolution that will enable the standardization of this therapeutic approach. Gil Villalba et al. [8] examines the potential of laser treatments, particularly fractional CO_2_ lasers, as an alternative therapy for Vulvar Lichen Sclerosus (VLS). VLS is a chronic inflammatory condition mainly affecting the anogenital area, frequently observed in postmenopausal women. Long-term, untreated cases of VLS can cause significant anatomical changes and lead to the development of squamous cell carcinoma. While high-potency topical corticosteroids remain the primary treatment, this review explores laser therapy’s role in managing the disease. This systematic review includes six controlled clinical trials involving 177 female patients. It compared laser treatments, particularly fractional CO_2_ lasers, with conventional corticosteroid therapies. The results show that although laser therapy reduced itching, pain and dyspareunia more effectively than corticosteroids in the short-term, no significant long-term histological differences were observed between the two treatments. The studies did note an increase in collagen production in the laser-treated group, suggesting tissue remodeling benefits. Moreover, patient satisfaction was higher with laser therapy, and its tolerability was excellent. The clinical importance of this study lies in the fact that while laser therapy shows promise in improving symptoms and patient satisfaction, the evidence is still insufficient to recommend it as a standalone treatment for VLS. However, combining laser therapy with corticosteroids may offer a synergistic approach to managing this condition. For daily clinical practice, this review highlights the potential for laser treatments to complement existing therapies, improving quality of life for patients unresponsive to conventional corticosteroid regimens.

In conclusion, this first edition synthesizes new dermatological evidence that concerns the essence of the pathophysiological mechanisms, clinical features, epidemiological factors and therapeutic advances of different entities, some of them with low prevalence (lichen sclerosus) and others more common (contact eczema), but which all share the need to continue deepening their knowledge.
